# AWSEM-Suite: a protein structure prediction server based on template-guided, coevolutionary-enhanced optimized folding landscapes

**DOI:** 10.1093/nar/gkaa356

**Published:** 2020-05-08

**Authors:** Shikai Jin, Vinicius G Contessoto, Mingchen Chen, Nicholas P Schafer, Wei Lu, Xun Chen, Carlos Bueno, Arya Hajitaheri, Brian J Sirovetz, Aram Davtyan, Garegin A Papoian, Min-Yeh Tsai, Peter G Wolynes

**Affiliations:** Department of Biosciences, Rice University, 6100 Main St, Houston, TX 77005, USA; Center for Theoretical Biological Physics, Rice University, 6100 Main St, Houston, TX 77005, USA; Center for Theoretical Biological Physics, Rice University, 6100 Main St, Houston, TX 77005, USA; Center for Theoretical Biological Physics, Rice University, 6100 Main St, Houston, TX 77005, USA; Center for Theoretical Biological Physics, Rice University, 6100 Main St, Houston, TX 77005, USA; Center for Theoretical Biological Physics, Rice University, 6100 Main St, Houston, TX 77005, USA; Department of Physics, Rice University, 6100 Main St, Houston, TX 77005, USA; Center for Theoretical Biological Physics, Rice University, 6100 Main St, Houston, TX 77005, USA; Department of Chemistry, Rice University, 6100 Main St, Houston, TX 77005, USA; Center for Theoretical Biological Physics, Rice University, 6100 Main St, Houston, TX 77005, USA; Department of Computer Science, University of Houston, 4800 Calhoun Rd, Houston, TX 77004, USA; Center for Theoretical Biological Physics, Rice University, 6100 Main St, Houston, TX 77005, USA; Center for Theoretical Biological Physics, Rice University, 6100 Main St, Houston, TX 77005, USA; Department of Chemistry, University of Maryland, College Park, MD 20742, USA; Department of Chemistry, Tamkang University, 151 Yingzhuan Road, New Taipei City 25137, Taiwan; Department of Biosciences, Rice University, 6100 Main St, Houston, TX 77005, USA; Center for Theoretical Biological Physics, Rice University, 6100 Main St, Houston, TX 77005, USA; Department of Physics, Rice University, 6100 Main St, Houston, TX 77005, USA; Department of Chemistry, Rice University, 6100 Main St, Houston, TX 77005, USA

## Abstract

The accurate and reliable prediction of the 3D structures of proteins and their assemblies remains difficult even though the number of solved structures soars and prediction techniques improve. In this study, a free and open access web server, AWSEM-Suite, whose goal is to predict monomeric protein tertiary structures from sequence is described. The model underlying the server’s predictions is a coarse-grained protein force field which has its roots in neural network ideas that has been optimized using energy landscape theory. Employing physically motivated potentials and knowledge-based local structure biasing terms, the addition of homologous template and co-evolutionary restraints to AWSEM-Suite greatly improves the predictive power of pure AWSEM structure prediction. From the independent evaluation metrics released in the CASP13 experiment, AWSEM-Suite proves to be a reasonably accurate algorithm for free modeling, standing at the eighth position in the free modeling category of CASP13. The AWSEM-Suite server also features a front end with a user-friendly interface. The AWSEM-Suite server is a powerful tool for predicting monomeric protein tertiary structures that is most useful when a suitable structure template is not available. The AWSEM-Suite server is freely available at: https://awsem.rice.edu.

## INTRODUCTION

Despite the continuing increase in the number of solved protein tertiary structures in the protein database, there remains a huge sequence-to-structure gap (1). While experimental techniques such as X-ray crystallography and cryo-electron microscopy grow in power, only a small fraction of the proteins out of the whole genome can be solved, due to the limitations of time and the extremely labor-intense nature of the laboratory work. Computational methods such as protein structure modeling and prediction are thus particularly useful, as these methods can complement results obtained from experimental methods.

The protein folding problem, in the sense of the underlying physics, has been solved by the energy landscape theory (2). The problem of matching the amino acid sequence to protein tertiary structure is however still challenging in a practical sense (3). There is already a significantly large coverage of motifs in solved protein structures, so much so that finding a novel structure domain is unusual nowadays (4). Template-based modeling, is growing in power but still requires templates that have sufficient sequence similarity (typically a >30% sequence identity is the minimum threshold), to be efficient and to generate a reliable model close to the native one (5). Recent improvements in co-evolutionary analysis along with deep learning algorithms with their origin in neural network theory, have allowed template-free methods to produce very often highly accurate models (6–8). This suggests that employing a variety of background data can indeed improve the performance of protein structure prediction over using purely physics.

Using standard sampling techniques, molecular dynamics simulation with all-atom force fields has been successfully applied to protein structure prediction and molecular docking (9,10). Simulations using fully atomistic models however are not very efficient in sampling. In any event, it appears all-atom models demand careful tuning in order to properly fold proteins (11). For molecular dynamics simulations accurate on biologically relevant timescales, a coarse-grained modeling framework can be used in which each amino-acid residue is represented by only a few extended force centers (12,13). The coarse-grained nature of this representation boosts the simulation timescales by several orders of magnitude. The Associative memory, Water-mediated, Structure and Energy Model (AWSEM)-Suite web server employs such a coarse-grained model to predict the 3D structure of proteins. The associative memory refers to the fragment memory term which imposes a local structural bias using short overlapping fragments. The model is based on the energy landscape theory of protein folding (3,14). The server’s coarse-grained protein model has been optimized both using physically motivated energies and knowledge based energy terms. The optimization scheme was inspired by work in neural network prediction of tertiary structure starting over 30 years ago (14). In contrast to the original versions of the associative memory prediction that mainly focused on constraining each peptide’s geometric properties and purely physical interactions at the short range (15), the current iteration of the algorithm has been augmented with long-range electrostatics interactions, template-based restraints and co-evolutionary information based restraints (5,16,17). The server’s predictions for protein 3D structure can be carried out using the contacts predicted from co-evolutionary signals (17), and/or experimentally determined contacts and may use partial structural data from a known 3D template of a homologous protein (5). In this paper, we describe the AWSEM-Suite server along with its component methods as needed by the user. The predictive power of AWSEM-Suite was demonstrated in the recent CASP experiments. The server also provides run-time visualization gadgets for the server’s input and output pages thus acting as a user-friendly interface. An illustrative example of job submission is provided in this article to demonstrate how the web server works.

## METHODS

### Server data processing

**Figure 1.**
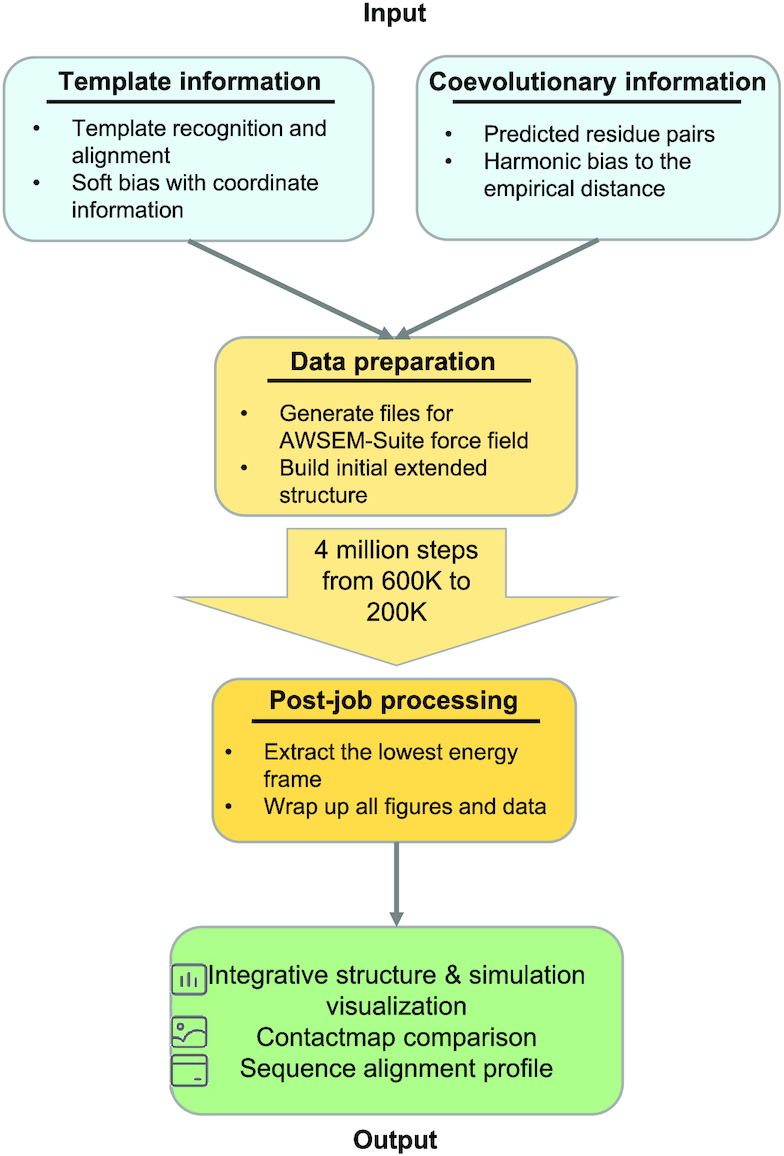
Flowchart of the AWSEM-Suite protocol.

AWSEM-Suite is built on the Large-Scale Atomic/Molecular Massively Parallel Simulator (LAMMPS) (18), an open source molecular dynamics simulation software. This server aims to provide users with a web-based interface to facilitate job submission. The overall workflow of AWSEM-Suite is illustrated in Figure 1. The physical energy terms of AWSEM are transferable and non-additive, meaning that the same protocol and parameters should work for all protein sequences. These terms maintain the geometry of backbone, and reflect the protein’s physical/chemical properties in the context of tertiary and secondary structure, as well as the intra- and inter-molecular hydrogen bonding profile of proteins. The details of the AWSEM energy terms can be found in previous work (15). In addition, the AWSEM-Suite server algorithm includes a template-based term and a co-evolutionary restraint term that greatly improves the predictive power. Users can carry out template-based modeling by providing multiple sequence alignment information or a single protein structure file as input. Co-evolutionary restraints can also be determined and applied by providing a contact list file that can be obtained from external servers. Additional contact information can also be added by the user, if it is available from experiments. In the simulations, a completely extended conformation of a given protein sequence is first generated. Starting from this conformation, dynamics is then followed by a 5 ns simulation using AWSEM-Suite to complete the initialization. Following the initialization, a period of annealing simulation is carried out. In this annealing step, the temperature is gradually lowered from high temperature (600 K) to low temperature (200 K) to obtain a stable structure. The three lowest energy frames from the annealing simulation are recorded. Additionally, users are allowed to submit multiple simulations at a single time, allowing them to either carry out a single simulation or multiple runs up to a maximum of five independent runs. The data from the jobs are stored in the server for one month.

### Software

The web application uses the C++ and the Python programming languages. The main code of AWSEM can be downloaded from (https://github.com/adavtyan/awsemmd). Several open source, on-line visualization tools are implemented in the AWSEM-Suite web application. The neXtProt protein sequence viewer (https://github.com/calipho-sib/sequence-viewer), NGL Viewer (http://nglviewer.org) and MSAviewer (https://msa.biojs.net) are used to visualize the input sequence information, the predicted structures and their annealing simulation trajectories, along with multiple sequence alignment information. These are shown in the results page (19).

## RESULTS AND DISCUSSION

### Input files for the server

On the job request page of AWSEM-Suite, a query sequence in FASTA file format is required to start to predict the three-dimensional structure. Additionally, the user may also choose to provide multiple sequence alignment (MSA) information in the HHR format from the HHpred website (https://toolkit.tuebingen.mpg.de/tools/hhpred) as indicated in the server’s documentation, which will then be used to choose a structural template with the lowest e-value for use as the structural restraint. If the user chooses a multiple sequence alignment file, any templates that have an *e*-value over 1 are excluded. The user can also upload a single PDB file as an input. The AWSEM-Suite server uses BLASTP to align the sequence of the template structure with the query sequence and then picks out the aligned region (20). Finally, the user may also choose to provide a file with co-evolutionary information, which contains predicted residue-residue contacts from either RaptorX-contact (http://raptorx.uchicago.edu/ContactMap/) or Gremlin (https://gremlin2.bakerlab.org/). These two web servers provide residue-residue contacts by analyzing inter-residue co-variation patterns in multiple sequence alignments (21–23). At this point, the user can augment the contact list with experimental data or, if desired, intuitive choices. If the user chooses not to provide the template and predicted contact files, the online server will generate the MSA file and the predicted contact file using the default settings of HHpred and MetaPSICOV respectively (19). An example of file submission can be found on the main page for reference.

The job submission page has two optional fields which a user can choose to fill in: a short job name and an email address, respectively. The user will receive a notification with the link in the beginning of the job submission and completion. If users do not wish to be notified via email, they can bookmark the link to the results page for later viewing.

### Server output result

**Figure 2.**
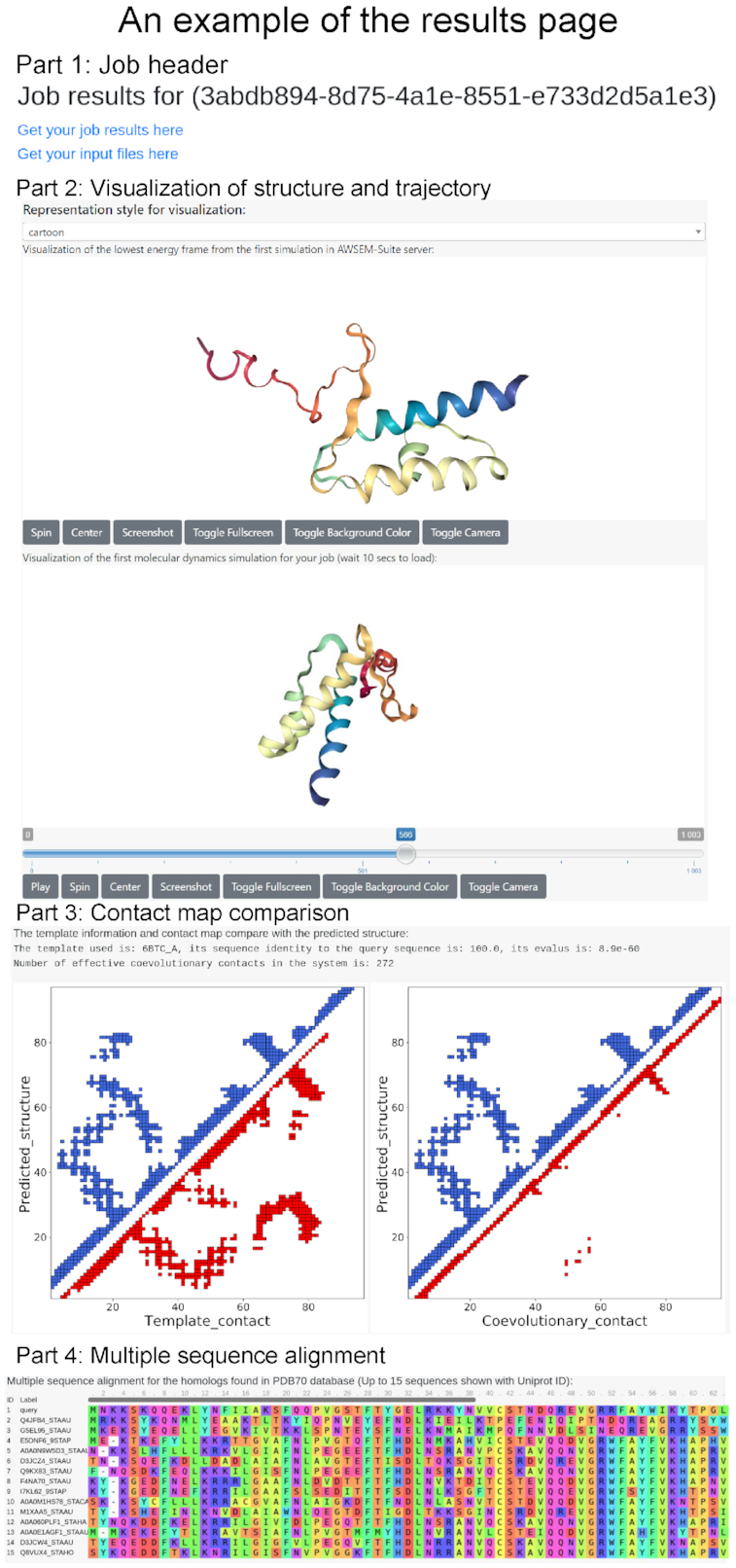
Example output page of AWSEM-Suite. The protein structure and the trajectory movies are visualized using NGL viewer. The models and trajectories are downloadable in PDB format. The input sequence and its homologs in the database are also shown in the page.

When a job is finished, the user will receive a link to the results page (given that an e-mail address is provided). An example of the graphical output of the AWSEM-Suite result page is shown in Figure 2. On top of the results page two links are found. The first link leads the user to the final results. The second link allows the user to download all of the input files in zip format, where one can check the sequence that was used in the simulation. The zip file contains several pdb files. The single pdb file that contains all the frames during the simulated annealing process is named with the prefix ‘trajectory’. Among all of the simulated frames, the lowest energy frame is named with the prefix ‘best’. Similarly, the three lowest energy frames that are extracted from those individual trajectories can be found by the trajectory index and the frame index. In addition, we provide the pdb file (*aligned.pdb*) of the aligned region that was used as the structural template in AWSEM-Suite. Also, two contact maps are attached. One map compares the contacts of the final predicted structures from the first simulation trajectory with the contact list that takes into account the coevolutionary terms. And the other map compares the contacts to those in the template structure. These contact maps are included in the results zip file with filename *template_contactmap.png* and *coer_contactmap.png*, respectively.

The results page shows the user’s input sequence for running the simulation as the first part. Then a 3D visualization of the structure corresponding to the lowest energy frame in the first trajectory of the user’s job presents itself in the next part. The structure is colored using a rainbow style with the blue region representing the N-terminus and the red region representing the C-terminus. The user can rotate the structure with the left mouse button, shift the structure with the right mouse button and zoom in and zoom out with the mouse wheel. A visualization of the first trajectory of the user’s job is shown in the next part. The user can drag the slide bar to choose the exact frame that is wanted and play the simulation movie.

The name, sequence identity and *e*-value of the selected template are shown. These will help to evaluate the quality of the template. The number of selected contacts in the coevolutionary contact file is also shown if the correct coevolutionary contact files were generated. The user can access two contact maps that compare the contacts of the final predicted structures of the first trajectory and the input template/coevolutionary contact list.

The bottom of the results page shows a multiple sequence alignment table visualized by the MSAviewer that includes several homologs found in the PDB70 database. The user can copy the label and go to Uniprot (https://www.uniprot.org) database to retrieve relevant biological information about each homolog.

### Independent benchmarking in CASP13

**Figure 3.**
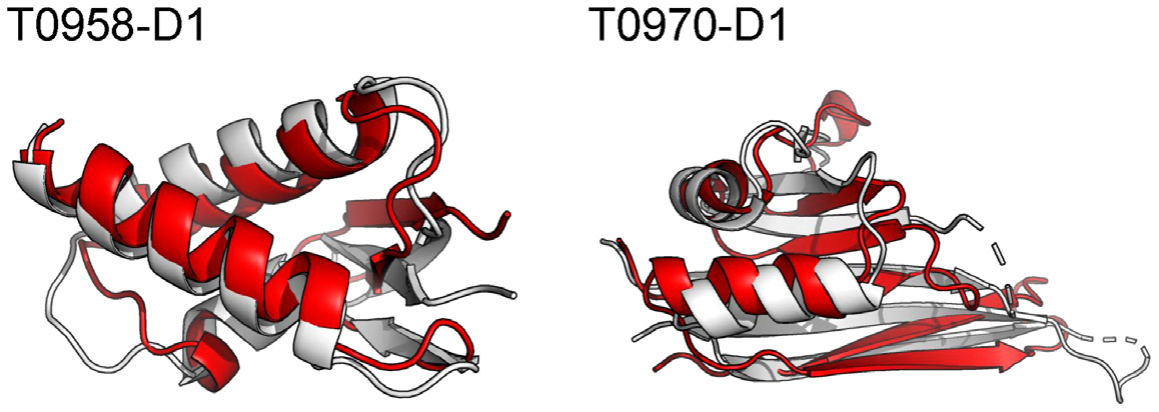
The structural alignment of the crystal structure of T0958-D1 and T0970-D1 that colored white and the theoretical model predicted by AWSEM-Suite that colored red. The RMSD value for the predicted structure of T0958-D1 is 3.48, for T0970-D1 is 4.61.

The biannual Critical Assessment of Structure Prediction (CASP) experiment provides an objective evaluation of state-of the-art methodologies in modeling protein structure from amino acid sequence (24). The AWSEM-Suite web server was tested by predicting the structure of 111 domains of 88 targets in CASP13 protein structure prediction competition, 21 of them belong to TBM-hard, 45 of them belong to TBM-easy, 32 of them belong to FM and the remaining belong to FM/TBM. These blind predictions were submitted to the competition by the group named ‘AWSEM-Suite’. Based on the modeling difficulty, domain sequences were divided into three categories during prediction: template-based modeling (TBM) for targets where one or more structure templates can be identified from sequence, free modeling (FM) for sequences without such templates, and the category TBM/FM which bridges these two categories (25). The AWSEM-Suite server ranked eighth among 39 groups in the TBM/FM category, and ranked 13th when all of the targets in the TBM-hard, TBM/FM and FM categories were combined. When judged by the performance in the TBM/FM category, we also found AWSEM-Suite to be comparable to other state-of-the-art servers evaluated by GDT-TS score. GDT is calculated as the fraction of the chain corresponding with the largest set of alpha carbon atoms in a structure falling within a defined distance cutoff of their position in another structure after superimposing them using the LGA algorithm (26). GDT-TS takes the average of cutoff of 2, 4, 6, 8 Å, ranges between 0 and 100 that represents similarity, with low values corresponding to bad correspondences. In the case of the target T0958-D1, AWSEM-Suite achieved the highest score among all entries, with a GDT-TS score 74.03. For the target T0970-D1, AWSEM-Suite submitted the second-best prediction with a GDT-TS score of 64.41. The GDT-TS values of these AWSEM-Suite predictions have a positive correlation to the percent of residues recognized as helix and strand in the crystal structure. The Pearson r-value of this tendency is 0.35. The performance of AWSEM-Suite and the state-of-the-art methods in all of the CASP13 TBM/FM category and FM category targets are listed in Table 1. Alignments of the server predicted structures with the corresponding crystal structures of T0958-D1 and T0970-D1 are shown in Figure 3. The authors note the AWSEM-Suite server is presently only able to predict monomeric proteins.

**Table 1.** GDT-TS value of the best predicted structures of AWSEM-Suite and other state-of-the-art methods for all the CASP13 TBM/FM and FM category targets

Domain ID	RaptorX-DeepModeller	Falcon	AWSEM-Suite	BAKER-ROSETTASERVER	Seok-server
All domains in FM/TBM category					
T0949-D1	65.31	62.98	55.81	N/A	57.75
T0953s2-D1	37.50	47.16	41.48	56.25	25.27
T0955-D1	71.95	95.12	64.02	N/A	47.56
T0958-D1	70.45	58.77	74.03	66.56	69.16
T0970-D1	55.00	34.41	64.41	57.65	21.77
T0978-D1	50.91	40.74	45.64	47.34	39.47
T0981-D3	51.97	36.08	34.85	42.61	8.25
T0986s1-D1	67.66	35.87	49.19	68.21	60.05
T0992-D1	69.86	38.78	63.78	81.78	47.20
T0997-D1	68.38	42.16	47.30	49.73	45.54
T1005-D1	54.76	49.54	42.33	55.83	47.09
T1008-D1	37.34	75.97	45.45	68.18	40.26
T1019s1-D1	61.64	40.95	40.52	83.62	76.72
All domains in FM category					
T0950-D1	38.52	20.83	19.66	33.26	21.64
T0953s1-D1	37.69	42.16	27.24	48.88	21.27
T0953s2-D2	63.96	43.92	61.26	45.27	14.19
T0953s2-D3	29.84	18.01	39.52	21.77	15.32
T0957s1-D1	34.72	35.19	35.42	39.35	20.83
T0957s2-D1	53.71	34.84	43.55	45.81	27.26
T0960-D2	51.19	28.87	35.42	56.55	19.94
T0963-D2	54.88	27.44	36.28	38.41	41.46
T0968s1-D1	57.63	33.48	44.49	66.74	24.79
T0968s2-D1	60.22	28.04	41.74	71.3	17.39
T0969-D1	46.05	23.02	23.38	30.3	29.17
T0975-D1	44.31	12.81	33.01	38.43	32.65
T0980s1-D1	46.88	30.29	32.69	40.14	22.84
T0981-D2	33.75	34.69	32.19	20.62	14.06
T0986s2-D1	48.23	27.9	36.77	25.32	20.48
T0987-D1	48.11	18.92	34.59	17.7	11.56
T0987-D2	41.71	25.89	19.13	23.98	9.82
T0989-D1	27.61	16.42	34.89	18.47	15.3
T0989-D2	38.17	23.21	23.88	26.56	16.3
T0990-D1	45.72	36.51	61.18	39.8	31.25
T0990-D2	24.89	14.07	27.38	18.61	12.88
T0990-D3	20.89	17.14	18.07	16.9	9.04
T0991-D1	25.68	24.32	24.77	35.36	22.3
T0998-D1	18.37	17.47	14.91	17.92	12.65
T1000-D2	49.59	8.63	14.13	58.83	59.44
T1001-D1	65.47	24.82	26.08	73.2	55.58
T1010-D1	22.62	9.05	10.95	29.76	12.86
T1015s1-D1	53.12	34.94	35.23	57.67	30.4
T1017s2-D1	59.4	31.6	23.4	59	21
T1021s3-D1	57.83	17.77	33.28	40.66	47.14
T1021s3-D2	57.73	34.28	29.9	25	19.59
T1022s-D1	51.44	18.27	22.76	34.78	24.04

## CONCLUSION

Under the guidance of energy landscape theory, the AWSEM-Suite server is able to quickly and effectively predict protein structures when a high sequence similarity template cannot be found. The AWSEM-Suite has been rigorously blind tested in the recent CASP13 prediction experiment where it ranked among the top 25% performing servers in FM and FM/TBM categories. The returned result pages from this server are designed to make available to non-expert users relevant prediction information in an intuitive manner with graphical assistance. The user-friendly interface design should be straightforward for most protein scientists to use. Given AWSEM’s proven track record and unique combination of an energy landscape optimized physical and bioinformatic model with template and co-evolutionary guidance, we feel that this web server will be useful to many protein scientists.
